# F12. INFLAMMATORY BIOMARKERS AND COGNITION IN FIRST EPISODE PSYCHOSIS: GENDER DIFFERENCES

**DOI:** 10.1093/schbul/sby017.543

**Published:** 2018-04-01

**Authors:** Bibiana Cabrera Llorca, Miquel Bioque, Gisela Mezquida, Ana Gonzalez-Pinto, Mara Parellada, Julio Bobes, Antonio Lobo, Borja García-Bueno, Karina MacDowell, Carla Torrent, Pilar Saiz-Martinez, Juan Carlos Leza, Miquel Bernardo

**Affiliations:** 1 Neurosciences Institute, Hospital Clinic of Barcelona; 2 Hospital Universitario Araba, Universidad del País Vasco; 3 Hospital General Universitario Gregorio Marañon, Universidad Complutense de Madrid; 4 University of Oviedo; 5 Instituto Investigación Sanitaria Aragón, University of Zaragoza; 6 Complutense University, Instituto de Investigación Hospital; 7 Universidad Complutense de Madrid; 8 Hospital Clinic Barcelona, Universitat Barcelona

## Abstract

**Background:**

Cognitive impairment is considered a central feature of psychotic disorders, with an important impact on prognosis and functional outcome (Nuechterlein et al., 2011). Among the etiological explanations on psychosis, several hypotheses involving alterations in the immune / inflammatory system have been proposed and recent research links these inflammatory processes with cognitive function, suggesting that the presence of inflammation is associated with poorer cognitive performance (Ribeiro-Santos et al., 2014, Cabrera et al., 2016). The study of the influence of gender on the possible association between inflammatory biomarkers and cognitive performance may favor the implementation of personalized treatments.

The aim of the study is to investigate the association between inflammatory biomarkers and gender-based cognition in a sample of first psychotic episode (FEP). A total of 92 patients with a FEP, 62 men and 30 women, recruited in five clinical centers were included. A neurocognitive assessment was performed and the expression of pro and anti-inflammatory mediators in peripheral blood mononuclear cells (PBMC) and plasma was measured.

**Methods:**

In the neurocognitive evaluation the domains of attention, verbal and working memory and executive function were included. Other and clinical variables included the assessment of psychotic and affective symptoms, age, sex, educational level and socio-economic level. The expression of the proinflammatory mediators (NFκB, iNOS, COX-2, PGE2, NO-2 and TBARS) and anti-inflammatory (15d-PGJ2, PPARγ and IκBα) of the main intracellular inflammatory pathway was measured in PBMC and plasma.

**Results:**

A correlation was made between inflammatory biomarkers and neurocognitive performance according to gender, and significant associations were selected to perform a subsequent hierarchical regression analysis. In the final model, only the expression of COX2 was associated with better performance in executive function in males (B = 0.504, p = <0.001) and the expression of NO2 to worse performance in working memory in women (B = -0.911; p = 0.010), after controlling the confounding factors. Men and women did not differ in any of the confusing variables.

**Discussion:**

The expression of certain pro / anti-inflammatory components could have a differential effect according to the gender of patients with FEP. The expression of COX2 could be beneficial in the case of males, explaining part of the variability in executive function. On the contrary, the expression of NO2 could have a detrimental effect on working memory in women. The establishment of biomarkers linked to gender-based cognition could be useful for monitoring the course of cognitive decline and could guide treatment programs, providing tools to select a personalized approach.

